# Application of machine learning techniques to the modeling of solubility of sugar alcohols in ionic liquids

**DOI:** 10.1038/s41598-023-39441-7

**Published:** 2023-07-27

**Authors:** Ali Bakhtyari, Ali Rasoolzadeh, Behzad Vaferi, Amith Khandakar

**Affiliations:** 1 Department of Chemical Engineering, Shiraz University, Shiraz, Iran; 2 Faculty of Engineering, Behbahan Khatam Alanbia University of Technology, Behbahan, Iran; 3 Department of Chemical Engineering, Shiraz Branch, Islamic Azad University, Shiraz, Iran; 4 Department of Advanced Calculations, Chemical, Petroleum, and Polymer Engineering Research Center, Shiraz Branch, Islamic Azad University, Shiraz, Iran; 5 Department of Electrical Engineering, Qatar University, Doha, 2713 Qatar

**Keywords:** Chemical engineering, Materials science

## Abstract

The current trend of chemical industries demands green processing, in particular with employing natural substances such as sugar-derived compounds. This matter has encouraged academic and industrial sections to seek new alternatives for extracting these materials. Ionic liquids (ILs) are currently paving the way for efficient extraction processes. To this end, accurate estimation of solubility data is of great importance. This study relies on machine learning methods for modeling the solubility data of sugar alcohols (SAs) in ILs. An initial relevancy analysis approved that the SA-IL equilibrium governs by the temperature, density and molecular weight of ILs, as well as the molecular weight, fusion temperature, and fusion enthalpy of SAs. Also, temperature and fusion temperature have the strongest influence on the SAs solubility in ILs. The performance of artificial neural networks (ANNs), least-squares support vector regression (LSSVR), and adaptive neuro-fuzzy inference systems (ANFIS) to predict SA solubility in ILs were compared utilizing a large databank (647 data points of 19 SAs and 21 ILs). Among the investigated models, ANFIS offered the best accuracy with an average absolute relative deviation (AARD%) of 7.43% and a coefficient of determination (R^2^) of 0.98359. The best performance of the ANFIS model was obtained with a cluster center radius of 0.435 when trained with 85% of the databank. Further analyses of the ANFIS model based on the leverage method revealed that this model is reliable enough due to its high level of coverage and wide range of applicability. Accordingly, this model can be effectively utilized in modeling the solubilities of SAs in ILs.

## Introduction

Biomass resources are a viable abundant, green, renewable, and sustainable alternative to conventional resources for chemical synthesis and energy delivery. Such a transition is intensified by reducing the amount of extractable fossil fuels, hardening environmental regulations, and stabilizing prices of biomass conversion^1–4^. The main avenues to achieving this milestone pass from the conversion of lignocellulosic biomass^5–7^. Since various substances can be synthesized by either direct or indirect lignocellulose conversion, among which sugars and sugar alcohols (SA) are of great interest^8–10^.

SAs, also known as polyols, are comprised of acyclic hydrogenated carbohydrates^11^. Thanks to their unique structures and the density of functional groups, SAs have found high popularity in pharmaceuticals, the food industry, and chemical processes^12^. Possessing similar or even better properties than conventional sugars, SAs also considered food ingredients^13^. Furthermore, they are increasingly utilized in pharmaceutical applications owing to their remarkable functional properties and health merits^14^. Despite existing in approximately small quantities, SAs were globally consumed up to 1.9 × 10^6^ metric tons in 2022^11,14^, which justifies the importance of developing reliable approaches to predict their properties and behavior. SA processing through biorefinery needs efficient solvents to pretreat or dissolve biomass, provide a suitable reaction medium, and enhance the conversion of sugars into either intermediates or ultimate products^15,16^.

To this end, many solvents with different characteristics, such as water, organic solvents, acids, bases, and ionic liquids (ILs)^17^ have been suggested. ILs not only offer liquid state and non-volatility in a broad range of temperatures but also benefit from high thermal stability and remarkable solubility strength. These characteristics make them potentially attractive tools to overcome various operational challenges^18^ associated with conventional solvents. The versatility of ILs allows their feature, thermochemical properties, and solvation power to be designed by adjusting the anion/cation pair appropriately^19–23^.

ILs offer high dissolving capacity for SAs due to the presence of various cations and anions, relatively low melting points, as well as ionic nature and non-volatility due to strong ionic-cationic interaction^24–29^. Xia et al. have recently reported the fabrication of cellulose- and lignin-obtained products employing ILs^30^. Accordingly, ILs have remarkable benefits in SAs extraction over conventional solvents. The solubilities of four sugar compounds (i.e., galactose, glucose, xylose, and fructose) in Aliquat®336 and 1-etyhl-3-methylimidazolium ethylsulfate ([Emim][EtSO_4_]) were measured (288–328 K) by Carneiro et al. and then correlated by two activity coefficient models (ACMs)^31^. Carneiro et al. also developed a theoretical and experimental study addressing the solubilities of sorbitol and xylitol in three ionic liquids in a wide range of temperatures (288–433 K) and evaluated some ACMs for thermodynamic modeling^32^. In another study, they measured the solubilities of sorbitol and xylitol in five different ILs namely 1-butyl-3-methylimidazolium dicyanamide ([Bmim][DCA]), 1-ethyl-3-methylimidazolium dicyanamide ([Emim][DCA]), 1-ethyl-3-methylimidazolium trifluoroacetate([Emim][TFA]), trihexyltetradecylphosphonium dicyanamide ([P_6,6,6,14_][DCA]), and Aliquat® dicyanamide at 288–339 K^33^. They also developed a thermodynamic model based on the perturbed-chain statistical associating fluid theory (PC-SAFT) equation of state (EoS). The solubility measurements of fructose and glucose in similar ILs were also done by the same research group^34^. Mohan et al. applied a molecular screening method based on the continuum solvation model to screen a large number of ILs for the solubility of xylose, glucose, fructose, and galactose over a somewhat wide temperature range (303.15 K to 373.15 K)^35^. They benefitted from the same approach to screen ILs for the solubility of sucrose, cellobiose, and maltose^36^. Paduszyński et al. measured solubility data and thermodynamic analysis of fructose, glucose, and sucrose in the presence of low-viscosity ILs composed of 1-butyl-3-methylimidazolium ([Bmim]^+^) cation and dicyanamide ([DCA]^−^) trifluoroacetate ([TFA]^−^) anions^37^. Their thermodynamic analysis was based on the PC-SAFT EoS. The same group investigated the solid–liquid equilibria of dicyanamide-based ILs and SAs (erythritol, xylitol, and sorbitol)^38^. A PC-SAFT modeling scheme was also employed to reproduce the measured data^38^. They also reported the impact of functionalized cations on the properties of ILs and their solubility strength for glucose^39^. The same thermodynamic approach utilizing the PC-SAFT approach was also developed in this system. The solubility of six monosaccharide SAs, namely glucose, mannose, fructose, galactose, xylose, and arabinose in different ILs composed of varied cations (1-butyl-3-methylimidazolium and trihexyltetradecylphosphonium) and anions (dicyanamide, dimethylphosphate, and chloride) were determined experimentally (288.2–348.2 K) and their solvation characteristics, as well as molecular-scale mechanisms, were studied by Teles et al.^40^. Asymmetric dicationic ILs have been recently introduced for this process and a pioneer study was developed by Yang et al., in which the impact of 1-(3-(trimethylammonio)prop-1-yl)-3-methylimidazolium bis(dicyanamide), 1-(3-(trimethylammonio)prop-1-yl)-1-methylpiperidinium bis(dicyanamide), and 1-(3-(trimethylammonio)prop-1-yl)pyridinium bis(dicyanamide) on the solubility of fructose and glucose was investigated at 323.15–353.15 K^41^. ACMs (Wilson, non-random two liquids (NRTL), and UNIQUAC) and semi-empirical equations (modified Apelblat and λh equation) were then applied to model the measured data. More recently, experimental investigations have addressed the solubility data of different compounds in numerous ILs^42–44^. Review studies also delve deeply into different aspects of this process^7,17,30^.

These thermodynamic-based calculations (i.e., semi-empirical equations, ACMs, and EoSs) are only applicable to a specific SA-IL system and it is not possible to use them for monitoring the phase equilibrium of several systems simultaneously. On the other hand, the artificial intelligence (AI) approaches can be simply applied to estimate the solubility of a wide range of SAs in different ILs. Hence, any effort leading to the simulating of the solubility of SAs in ILs with the use of machine learning (ML) tools is currently of great interest. ML-based tools have already been engaged in the accurate, fast, and easy-to-use estimation of the equilibrium data^20,45–49^, process assessment^50,51^, the properties of solvents^22,52–56^, oil reservoirs^57^, gas shales^58^, and biomass-derived materials^59^.

The solubility of SAs in ILs is a strong function of temperature and ILs type, which is identified by their properties. The type and properties of the SA compound also affect the solvation behavior. Early thermodynamic models utilizing EoSs^33,38,39^ and ACMs^31,32,34,35^ have several disadvantages. To elaborate on this, these models can accurately calculate solubility data only over a narrow and limited range of conditions. Moreover, they are component-specific, which results in the generation of too many parameters for various SA-IL systems. Consequently, a large number of component-specific parameters are found in the literature. On account of this, a universal ML model capable of covering a wide range of SA-IL systems, and temperatures is of paramount interest. From different standpoints, if the application of the ML models gains success, these models can possibly replace the conventional computation methods because of their facile usability and short computation period. ML is now widely utilized to address engineering issues, such as thermophysical property estimation^60,61^.

To benefit from a broad application of sugar alcohols in pharmaceuticals, the food industry, and chemical processes, it is necessary to extract them first. Feasible study, design, and optimization of SA extraction by ionic liquids require precise knowledge about the solubility data. Since the experimental measurement of SAs solubility in ILs is time-consuming and the literature introduces no comprehensive model for its estimation, the present study applies artificial intelligence tools for the considered task. The constructed intelligent model in this study can effectively engage in the simulation and optimization of the SA extraction by ILs.

## Theory

### Relevancy analysis

Before explaining ML models, it is essential to identify the direction and strength of the relationship between the system variables, namely solubility as the dependent variable and temperature, and SA and IL types, which are independent variables. This can be managed by facile and easy-to-interpret statistical analyses that measure the monotonic association between independent-dependent pairs of variables^62^. Pearson, Spearman, and Kendall's analyses benefit from a statistical notion known as covariance, which signifies the degree of correlation or the strength of the relationship between two variables. In other words, these analyses offer a straightforward criterion for how a pair of variables vary together^63,64^.

Pearson’s analysis [Eq. (1)] introduces a dimensionless parameter (− 1 to + 1) and Spearman’s criterion [Eq. (2)] offers the same range of the criterion and is actually the modified version of Pearson’s equation^62^. Even though the range of Spearman’s and Pearson’s parameters is the same, their quantitative and qualitative prediction of a single independent-dependent pair may differ^62^. For a system composed of a set of input (X) and output (Y) variables the Pearson (r) and Spearman (${\mathrm{r}}^{{^{\prime}}}$) coefficients can be calculated as follows:

1 $$\mathrm{r}=\frac{\sum_{\mathrm{i}=1}^{\mathrm{NDP}}\left({\mathrm{X}}_{\mathrm{i}}-{\mathrm{X}}_{\mathrm{av}}\right)\left({\mathrm{Y}}_{\mathrm{i}}-{\mathrm{Y}}_{\mathrm{av}}\right)}{\sqrt{\sum_{\mathrm{i}=1}^{\mathrm{NDP}}{\left({\mathrm{X}}_{\mathrm{i}}-{\mathrm{X}}_{\mathrm{av}}\right)}^{2}}\sqrt{\sum_{\mathrm{i}=1}^{\mathrm{NDP}}{\left({\mathrm{Y}}_{\mathrm{i}}-{\mathrm{Y}}_{\mathrm{av}}\right)}^{2}}}$$

2 $${\mathrm{r}}^{{^{\prime}}}=1-\frac{6\sum_{\mathrm{i}=1}^{\mathrm{NDP}}{\mathrm{d}}_{\mathrm{i}}^{2}}{{\mathrm{NDP}}^{3}-\mathrm{NDP}}$$

where NDP and d indicate the number of data points and the difference between the two ranks of each observation, correspondingly. Kendall’s criterion [Eq. (3)] benefits from a correlation coefficient that is based on the ranks of the observations^64,65^.

3 $${\mathrm{r}}^{{^{\prime}}{^{\prime}}}=\frac{2\left({\mathrm{N}}_{\mathrm{c}}-{\mathrm{D}}_{\mathrm{d}}\right)}{{\mathrm{NDP}}^{2}-\mathrm{NDP}}$$

By employing the correlation parameters (r, ${\mathrm{r}}^{{^{\prime}}}$, and ${\mathrm{r}}^{{^{\prime}}{^{\prime}}}$), the relationship between the dependent variable (SAs solubility in ILs) and the independent variables (temperature, the molecular weights (MW) of SA and IL, the density of IL, and the fusion temperature and enthalpy) can be determined based on the regulations presented in Table 1.

**Table 1:** Range and interpretation of the correlation parameters^62^.

Range	Direction of change	Relationship
− 1.00 to − 0.90	Reverse change	Very strong
− 0.89 to − 0.70		Strong
− 0.69 to − 0.40		Moderate
− 0.39 to − 0.10		Weak
− 0.10 to − 0.00		Negligible
0.00	No impact	No inter-relation
0.00 to + 0.10	Direct change	Negligible
+ 0.10 to + 0.39		Weak
+ 0.40 to + 0.69		Moderate
+ 0.70 to + 0.89		Strong
+ 0.90 to + 1.00		Very strong

The main idea of this study lies in the use of ML models with the simplest procedure. Other than that, the employed parameters can simply represent the nature of the materials in question and distinguish them, since the temperature is the main effective process variable in SA-IL solid–liquid equilibria; MWs can distinguish the compounds and somewhat representative of the molecular length; Fusion enthalpy and temperature are characteristics of the solubility of solids in liquids; And the density of IL shed light on the solubility power of the solvent (IL). These variables are easily available for the entire databank. Other variables mentioned by the reviewer are not available for all the whole compounds, and vaporization temperature does not make sense in this system as the evaporation of ILs is infinitesimal. On account of such a standpoint, these variables were selected for modeling the systems in question.

These analyses and further analysis of the ML models are implemented based on a solubility databank (647 data points of 19 SAs and 21 ILs). The databank is reported in Table 2. The properties of ILs and SAs are also summarized in Tables 3 and 4, respectively.

**Table 2:** The experimental data collected from the literature to develop machine-learning models.

Sugar alcohol	Ionic liquid		NDP^a^	Solubility^b^	Temperature (K)	Reference
	Name	Abbreviation				
Xylitol	1-Ethyl-3-methyl-imidazolium dicyanamide	[EMIM][DCA]	7	0.1656–0.3835	288.30–327.80	^33^
	1-Butyl-3-methyl-imidazolium dicyanamide	[BMIM][DCA]	14	0.1380–0.8890	288.20–362.18	^33,66^
	1-Ethyl-3-methylimidazolium trifluoroacetate	[EMIM][TFA]	5	0.2943–0.4500	289.38–329.10	^33^
	1-Ethyl-3-methylimidazolium ethyl sulfate	[EMIM][EtSO_4_]	5	0.1652–0.4110	289.20–328.36	^32^
	1,3-Dimethylimidazolium dicyanamide	[C_1_C_1_IM][N(CN)_2_]	8	0.2157–0.9139	305.60–362.92	^38^
	1-(2-Hydroxyethyl)-3-methylimidazolium dicyanamide	[C_2_OHC_1_IM][N(CN)_2_]	11	0.0946–0.9002	290.50–362.52	^38^
	1-(3-Cyanopropyl)-3-methylimidazolium dicyanamide	[C_3_CNC_1_IM][N(CN)_2_]	7	0.2858–0.9183	327.50–363.70	^38^
	1-Ethyl-3-methylimidazolium 2-(2-methoxyethoxy)ethylsulfate	[EMIM][MeOEtOEtSO_4_]	6	0.0400–0.7537	305.72–357.46	^67^
	1-Butyl-3-methylimidazolium hydrogen sulfate	[BMIM][HSO_4_]	6	0.0338–0.6083	325.35–368.46	^67^
	1-Butyl-3-methylimidazolium thiocyanate	[BMIM][SCN]	6	0.0258–0.8154	294.44–359.10	^67^
	1-Butyl-3-methylimidazolium tricyanomethanide	[BMIM][C(CN)_3_]	6	0.0299–0.5021	333.89–362.63	^67^
Sorbitol	1-Ethyl-3-methyl-imidazolium dicyanamide	[EMIM][DCA]	5	0.2067–0.3697	288.30–328.30	^33^
	1-Butyl-3-methyl-imidazolium dicyanamide	[BMIM][DCA]	15	0.1745–0.7660	297.35–367.61	^33,66^
	1-Ethyl-3-methylimidazolium trifluoroacetate	[EMIM][TFA]	5	0.2579–0.4207	288.50–325.83	^33^
	1-Ethyl-3-methylimidazolium ethyl sulfate	[EMIM][EtSO_4_]	4	0.2449–0.3981	299.45–327.00	^32^
	1,3-Dimethylimidazolium dicyanamide	[C_1_C_1_IM][N(CN)_2_]	9	0.1295–0.9204	293.60–368.59	^38^
	1-(2-Hydroxyethyl)-3-methylimidazolium dicyanamide	[C_2_OHC_1_IM][N(CN)_2_]	9	0.1977–0.8963	313.72–369.44	^38^
	1-(3-Cyanopropyl)-3-methylimidazolium dicyanamide	[C_3_CNC_1_IM][N(CN)_2_]	9	0.1760–0.9195	315.40–370.47	^38^
	1-(2-Chloroethyl)-3-methylimidazolium dicyanamide	[C_2_ClC_1_IM][N(CN)_2_]	8	0.1645–0.9056	304.34–369.34	^38^
	1,3-Dimethylimidazolium dimethyl phosphate	[MMIM](MeO)_2_PO_2_	4	0.3480–0.6172	299.14–337.29	^42^
	1-Ethyl-3-methyl-imidazolium acetate	[C_2_MIM][Ac]	4	0.5180–0.6540	303.15–331.73	^43^
Erythritol	1,3-dimethylimidazolium dicyanamide	[C_1_C_1_IM][N(CN)_2_]	9	0.1351–0.9199	309.31–387.92	^38^
	1-(2-Hydroxyethyl)-3-methylimidazolium dicyanamide	[C_2_OHC_1_IM][N(CN)_2_]	14	0.1367–0.9527	319.10–389.30	^38^
	1-(3-Cyanopropyl)-3-methylimidazolium dicyanamide	[C_3_CNC_1_IM][N(CN)_2_]	10	0.1690–0.9355	324.23–388.70	^38^
	1-(2-Chloroethyl)-3-methylimidazolium dicyanamide	[C_2_ClC_1_IM][N(CN)_2_]	10	0.1147–0.9104	309.50–387.70	^38^
Mannitol	1-Ethyl-3-methylimidazolium 2-(2-methoxyethoxy)ethylsulfate	[EMIM][MeOEtOEtSO_4_]	4	0.0369–0.2974	330.22–389.06	^67^
	1-Butyl-3-methylimidazolium hydrogen sulfate	[BMIM][HSO_4_]	5	0.0271–0.2770	337.41–388.42	^67^
	1-Butyl-3-methylimidazolium thiocyanate	[BMIM][SCN]	6	0.0195–0.2141	305.09–368.42	^67^
	1-Butyl-3-methylimidazolium tricyanomethanide	[BMIM][C(CN)_3_]	4	0.0250–0.0926	380.91–411.42	^67^
Glucose	1-Ethyl-3-methylimidazolium ethyl sulfate	[EMIM][EtSO_4_]	5	0.1321–0.2895	271.14–326.84	^31^
	1-Ethyl-3-methylimidazolium trifluoroacetate	[EMIM][TFA]	5	0.2610–0.3490	284.20–326.30	^34^
	1-Ethyl-3-methyl-imidazolium dicyanamide	[EMIM][DCA]	5	0.1710–0.2810	288.25–325.63	^34^
	1,3-Dimethylimidazolium dicyanamide	[C_1_C_1_Im][N(CN)_2_]	5	0.1618–0.5805	316.50–391.14	^39^
	1-(2-Hydroxyethyl)-3-methylimidazolium dicyanamide	[C_2_OHC_1_Im][N(CN)_2_]	6	0.2205–0.5367	323.37–385.77	^39^
	1-(3-Cyanopropyl)-3-methylimidazolium dicyanamide	[C_3_CNC_1_Im][N(CN)_2_]	6	0.0978–0.5008	321.22–386.01	^39^
	1-(2-Chloroethyl)-3-methylimidazolium dicyanamide	[C_2_ClC_1_Im][N(CN)_2_]	5	0.1939–0.5841	332.54–394.40	^39^
	1-Butyl-3-methylimidazolium dimethylphosphate	[C_4_C_1_IM][(OCH_3_)_2_PO_4_]	6	0.0350–0.2320	288.20–335.32	^40^
	1-Butyl-3-methyl-imidazolium dicyanamide	[BMIM][DCA]	22	0.1330–0.7660	283.96–406.04	^34,37,40^
	1-Ethyl-3-methylimidazolium thiocyanate	[EMIM][SCN]	13	0.0566–0.3851	268.01–368.94	^35,68^
	1-Butyl-3-methylimidazolium trifluoroacetate	[C_4_C_1_IM][CF_3_CO_2_]	9	0.1610–0.8890	289.14–361.30	^37^
	1-(3-(Trimethylammonio)prop-1-yl)-3-methylimidazolium bis(dicyanamide)	[MIMC_3_N_111_][N(CN)_2_]_2_	7	0.4783–0.6425	307.62–361.89	^41^
	1-Ethyl-3-methylimidazolium 2-(2-methoxyethoxy)ethylsulfate	[EMIM][MeOEtOEtSO_4_]	4	0.0406–0.4691	328.33–384.71	^67^
	1-Butyl-3-methylimidazolium hydrogen sulfate	[BMIM][HSO_4_]	9	0.0261–0.5694	350.90–394.36	^67^
	1-Butyl-3-methylimidazolium thiocyanate	[BMIM][SCN]	5	0.0219–0.5227	300.77–386.56	^67^
	1-Butyl-3-methylimidazolium tricyanomethanide	[BMIM][C(CN)_3_]	4	0.0253–0.1834	362.18–398.76	^67^
	1,3-Dimethyl-imidazolium methyl phosphonate	[DMIM][MPh]	9	0.0532–0.6154	285.15–329.26	^68^
	1-Butyl-3-methylimidazolium chloride	[BMIM][Cl]	8	0.0573–0.4923	343.15–384.34	^68^
	1-Ethanol-3-methylimidazolium chloride	[EtOHMIM][Cl]	9	0.1096–0.5549	355.15–392.05	^68^
Galactose	1-Ethyl-3-methylimidazolium ethyl sulfate	[EMIM][EtSO_4_]	5	0.0518–0.1759	284.21–328.20	^31^
	1-Ethyl-3-methylimidazolium thiocyanate	[EMIM][SCN]	6	0.0410–0.1641	303.15–350.76	^35^
	1-Butyl-3-methyl-imidazolium dicyanamide	[BMIM][DCA]	7	0.1420–0.2980	286.57–347.20	^40^
Cellobiose	1-Ethyl-3-methylimidazolium thiocyanate	[EMIM][SCN]	6	0.0480–0.1436	290.34–353.15	^36^
Maltose	1-Ethyl-3-methylimidazolium thiocyanate	[EMIM][SCN]	6	0.1266–0.4280	304.15–355.10	^36^
Mannose	1-Butyl-3-methyl-imidazolium dicyanamide	[BMIM][DCA]	7	0.1720–0.3900	287.61–348.85	^40^
	1-Butyl-3-methylimidazolium dimethylphosphate	[C_4_C_1_IM][(OCH_3_)_2_PO_4_]	6	0.0290–0.2300	280.24–336.31	^40^
Fructose	1-Ethyl-3-methylimidazolium ethyl sulfate	[EMIM][EtSO_4_]	5	0.3121–0.5024	288.20–329.30	^31^
	1-Ethyl-3-methylimidazolium trifluoroacetate	[EMIM][TFA]	3	0.3550–0.4150	288.05–308.30	^34^
	1-Ethyl-3-methyl-imidazolium dicyanamide	[EMIM][DCA]	5	0.3330–0.4950	289.95–328.96	^34^
	1-Butyl-3-methylimidazolium trifluoroacetate	[C_4_C_1_IM][CF_3_CO_2_]	11	0.2050–0.8820	313.80–372.95	^37^
	1-Butyl-3-methyl-imidazolium dicyanamide	[BMIM][DCA]	22	0.1100–0.7490	295.60–369.83	^34,37,40^
	1-Ethyl-3-methylimidazolium thiocyanate	[EMIM][SCN]	14	0.0417–0.7136	281.15–361.14	^35,69^
	1-Butyl-3-methylimidazolium dimethylphosphate	[C_4_C_1_IM][(OCH_3_)_2_PO_4_]	6	0.0150–0.1250	280.16–337.20	^40^
	1-(3-(Trimethylammonio)prop-1-yl)-3-methylimidazolium bis(dicyanamide)	[MIMC_3_N_111_][N(CN)_2_]_2_	7	0.6068–0.7432	320.79–349.50	^41^
	1,3-Dimethyl-imidazolium methyl phosphonate	[DMIM][MPh]	9	0.0532–0.6154	284.95–353.63	^69^
	1-Butyl-3-methylimidazolium chloride	[BMIM][Cl]	9	0.0486–0.4923	339.15–358.15	^69^
	1-Ethanol-3-methylimidazolium chloride	[EtOHMIM][Cl]	9	0.0912–0.5752	352.00–366.50	^69^
Xylose	1-Ethyl-3-methylimidazolium ethyl sulfate	[EMIM][EtSO_4_]	5	0.2174–0.3728	288.10–324.73	^31^
	1-Ethyl-3-methylimidazolium thiocyanate	[EMIM][SCN]	6	0.1113–0.3310	303.15–351.09	^35^
	1-Butyl-3-methyl-imidazolium dicyanamide	[BMIM][DCA]	7	0.1820–0.4020	288.20–347.70	^40^
	1-Butyl-3-methylimidazolium dimethylphosphate	[C_4_C_1_IM][(OCH_3_)_2_PO_4_]	6	0.0800–0.5030	288.20–333.08	^40^
	1-Ethyl-3-methylimidazolium 2-(2-methoxyethoxy)ethylsulfate	[EMIM][MeOEtOEtSO_4_]	4	0.0425–0.4686	325.00–383.47	^67^
	1-Butyl-3-methylimidazolium hydrogen sulfate	[BMIM][HSO_4_]	7	0.0311–0.6115	349.58–392.09	^67^
	1-Butyl-3-methylimidazolium thiocyanate	[BMIM][SCN]	5	0.0261–0.5191	288.93–379.29	^67^
	1-Butyl-3-methylimidazolium tricyanomethanide	[BMIM][C(CN)_3_]	4	0.0466–0.2763	343.99–390.04	^67^
Lactose	1-Ethyl-3-methylimidazolium thiocyanate	[EMIM][SCN]	7	0.0122–0.0818	276.47–381.73	^69^
	1,3-Dimethyl-imidazolium methyl phosphonate	[DMIM][MPh]	8	0.0273–0.1809	272.75–346.58	^69^
	1-Butyl-3-methylimidazolium chloride	[BMIM][Cl]	6	0.0126–0.1251	344.15–382.68	^69^
	1-Ethanol-3-methylimidazolium chloride	[EtOHMIM][Cl]	6	0.0117–0.1062	349.50–382.95	^69^
Sucrose	1-Ethanol-3-methylimidazolium chloride	[EtOHMIM][Cl]	9	0.0244–0.2405	356.15–386.32	^69^
	1-Butyl-3-methylimidazolium chloride	[BMIM][Cl]	9	0.0262–0.2538	343.95–387.10	^69^
	1-Ethyl-3-methylimidazolium 2-(2-methoxyethoxy)ethylsulfate	[EMIM][MeOEtOEtSO_4_]	4	0.0191–0.2349	343.60–403.80	^67^
	1-Butyl-3-methylimidazolium thiocyanate	[BMIM][SCN]	6	0.0116–0.2392	315.20–403.75	^67^
	1-Butyl-3-methylimidazolium tricyanomethanide	[BMIM][C(CN)_3_]	3	0.0067–0.0361	381.20–406.64	^67^
	1-Butyl-3-methyl-imidazolium dicyanamide	[BMIM][DCA]	8	0.1880–0.7530	318.88–359.59	^37^
	1-Butyl-3-methylimidazolium trifluoroacetate	[C_4_C_1_IM][CF_3_CO_2_]	9	0.1920–0.8680	306.73–360.74	^37^
	1,3-Dimethyl-imidazolium methyl phosphonate	[DMIM][MPh]	8	0.0287–0.3595	262.47–355.24	^69^
	1-Butyl-3-methylimidazolium hydrogen sulfate	[BMIM][HSO_4_]	6	0.0139–0.2710	358.74–377.26	^67^
	1-Ethyl-3-methylimidazolium thiocyanate	[EMIM][SCN]	14	0.0125–0.1749	274.13–353.97	^36,69^
Total			647			

^a^Number of data points, ^b^Molar fraction.

**Table 3:** The properties of ionic liquids utilized in machine-learning models.

Ionic liquid	Molecular weight (kg/kmol)	Density (g/cm^3^)	Source
[EMIM][DCA]	177.21	1.108	^70^
[BMIM][DCA]	205.26	1.060	^71^
[EMIM][TFA]	224.18	1.291	^72^
[EMIM][EtSO_4_]	236.29	1.236	^73^
[C_1_C_1_IM][N(CN)_2_]	189.22	1.109	^39^
[C_2_OHC_1_IM][N(CN)_2_]	193.21	1.095	*
[C_3_CNC_1_IM][N(CN)_2_]	216.24	1.271	*
[C_2_ClC_1_IM][N(CN)_2_]	211.65	1.238	^39^
[EMIM][MeOEtOEtSO_4_]	310.37	1.168	*
[BMIM][HSO_4_]	236.29	1.220	^74^
[BMIM][SCN]	197.30	1.070	^75^
[BMIM][C(CN)_3_]	229.28	1.059	*
[MMIM](MeO)_2_PO_2_	222.18	1.265	^42^
[C_2_MIM][Ac]	170.21	1.099	^76^
[EMIM][SCN]	169.25	1.116	^75^
[C_4_C_1_IM][CF_3_CO_2_]	252.24	1.212	^77^
[C_4_C_1_IM][(OCH_3_)_2_PO_4_]	264.26	1.160	^78^
[MIMC_3_N_111_][N(CN)_2_]_2_	315.38	1.153	^41^
[DMIM][MPh]	192.15	1.068	*
[BMIM][Cl]	174.67	1.074	^79^
[EtOHMIM][Cl]	162.62	1.331	*

*Estimated by group contribution methods^26,80,81^.

**Table 4:** The properties of sugar alcohols utilized in machine-learning models.

Sugar alcohol	Molecular weight (kg/kmol)	Density (g/cm^3^)	Fusion temperature (K)	Enthalpy of fusion (kJ/kmol)	Source
Xylitol	152.146	1.515	365.70	37,400	^32,82^
Sorbitol	182.171	1.489	366.50	30,200	^32,82,83^
Erythritol	122.120	1.451	390.90	39,400	^82–84^
Mannitol	182.171	1.489	439.10	56,100	^82,83^
Glucose	180.155	1.562	423.15	32,432	^83,85^
Fructose	180.155	1.665	378.15	26,030	^83,85^
Sucrose	342.296	1.580	459.15	46,187	^83,85^
Xylose	150.130	1.525	416.15	31,700	^83,84^
Galactose	180.160	1.500	436.15	43,800	^84^
Cellobiose	342.300	1.415	495.15	31,058	^36^
Maltose	360.312	1.768	377.15	45,400	^84^
Mannose	180.155	1.539	407.15	24,687	^83,86^
Lactose	342.296	1.590	474.15	75,306	^83,86^

### Machine learning

Artificial neural networks (ANN) have different variants, including multilayer perceptron (MLP), radial basis function (RBF), recurrent neural networks (RNN), general regression neural network (GRNN), and cascade feed-forward neural network (CFFNN)^87,88^. The smallest meaningful section of the ANN is the artificial neurons, which are assigned to performing calculations based on Eq. (4)^89^.

4 $$\mathrm{z}=\mathrm{\varphi }[\left(\sum_{\mathrm{i}=1}^{\mathrm{n}}{\mathrm{x}}_{\mathrm{i}}\times {\mathrm{w}}_{\mathrm{i}}\right)+\mathrm{b}]$$

In Eq. (4), z stands for the neuron’s output; while a particular artificial neuron received n inputs (${\mathrm{x}}_{\mathrm{i}}$), and each connection is adjusted by a corresponding weight (${\mathrm{w}}_{\mathrm{i}}$). Moreover, each neuron contains one extra adjusting parameter, which is called bias ($\mathrm{b}$). To overcome the restriction of only linear input–output mappings and propose a strategy to model nonlinear relationships, a frequently nonlinear activation function ($\mathrm{\varphi }$) is also incorporated in the neuron body. Linear, hyperbolic, tangent, logistic, and Gaussian activation functions can be implemented in the neuron structure^90^.

All of the ANN models include three types of layers: an input layer that receives the independent variables, the output layer that delivers the target prediction, and single or multiple hidden layers which have the task of data processing and recognition^90^. The number of independent features and dependent variables determines the number of elements in the input and output layers, respectively.

The training phase of ANN is responsible for obtaining appropriate values of the bias/weight that provide the best prediction accuracy for a dependent variable. This study applied the following ANN models to find the best one in the calculation of SAs solubilities in a variety of ILs.

#### MLP neural network (MLPNN)

This model utilizes a supervised learning technique called backpropagation for training and is a reliable model in many modeling fields. This study equips the hidden and output layers of the MLP model with the tangent hyperbolic [Eq. (5)] and logarithm sigmoid [Eq. (6)] activation functions, respectively^90^.

5 $${\varphi }\left(\mathrm{Z}\right)=\frac{1-\mathrm{exp}(-2\mathrm{Z})}{1+\mathrm{exp}(-2\mathrm{Z})}$$

6 $${\varphi }\left(\mathrm{Z}\right)=\frac{1}{1+\mathrm{exp}(-\mathrm{Z})}$$

#### RBF network (RBFN)

This model utilizes Gaussian or RBF as the activation function [Eq. (7)^64^] in the hidden layer, whereas its output is a linear combination of neuron parameters and RBF transformation of the inputs [Eq. (8)]. One of the best features of this model is its simplicity and fast-training nature^47^.

7 $${\varphi }\left(\mathrm{Z}\right)=\mathrm{exp}(\frac{{\mathrm{Z}}^{2}}{2{\upsigma }^{2}})$$

8 $${\varphi }\left(\mathrm{Z}\right)=\mathrm{Z}$$

where $\upsigma$ is the spread factor.

To obtain the best RBF performance, the number of nodes in the hidden layer and the spread coefficient must determine carefully^47^.

#### Cascade feed-forward neural network (CFFNN)

This model generates a cascade configuration that links the nodes of the input to the hidden and output layers^46^. This model also utilizes the tangent hyperbolic and logarithm sigmoid transfer functions in the hidden and output layers, respectively.

It is worth noting that the learning step alters the connection weights and biases by a predefined optimization algorithm. This optimization algorithm continuously changes the model’s weights and biases to minimize the prediction error between the model output and the expected target (real data). This study employs the Levenberg–Marquardt (LM) algorithm to accomplish the training phase of the CFF and MLP models.

#### General regression neural network (GRNN)

Similar to the MLP and CFFNN, this ANN type also constitutes of the input, hidden, and output layers. The last two layers have the Gaussian and linear transfer functions, respectively. The only difference between GRNN and RBFN topologies is that the number of hidden neurons of the earlier is fixed and cannot be manipulated^91^.

#### Adaptive neuro-fuzzy inference systems (ANFIS)

ANFIS is designed by combining fuzzy logic and ANN to benefit from the strength of both models. This model consists of five successive layers, namely the first layer (fuzzy formation), the second layer (fuzzy rules), the third layer (normalization of membership functions), the fourth layer (fuzzy rule conclusion section), and the fifth layer (output calculation). By minimizing the observed error between the predicted and actual responses utilizing an appropriate scenario, the parameters of ANFIS can be adjusted^92^.

#### Least-squares support vector regression (LSSVR)

This method is capable of transferring the independent variables to a multi-dimensional space through the application of kernel functions (K)^92^. The most well-known and widely-employed kernel types that are employed in the LSSVR model are polynomial, linear, and Gaussian.

### Uncertainty criteria

To check the reliability of the models, coefficient of determination (R^2^), mean squared error (MSE), root-mean-square deviation (RMSE), average absolute relative deviation percentage (AARD), mean absolute error (MAE), and relative absolute error (RAE), which are presented in Eqs. (9–14), are assessed. These variables are then employed in ranking the models.

9 $${R}^{2}=1- \left(\frac{\sum_{\mathrm{i}=1}^{\mathrm{NDP}}{({\mathrm{X}}_{\mathrm{i}}^{\mathrm{calc}.}-{\mathrm{X}}_{\mathrm{i}}^{\mathrm{exp}.})}^{2}}{\sum_{\mathrm{j}=1}^{\mathrm{NDP}}{({\mathrm{X}}_{\mathrm{j}}^{\mathrm{exp}.}-{\bar{\mathrm{X}} }^{\mathrm{exp}.})}^{2}} \right)$$

10 $${\mathrm{MSE}}=\frac{1}{\mathrm{NDP}}\sum_{\mathrm{j}=1}^{\mathrm{NDP}}{({\mathrm{X}}_{\mathrm{j}}^{\mathrm{calc}.}-{\mathrm{X}}_{\mathrm{j}}^{\mathrm{exp}.})}^{2}$$

11 $${\mathrm{RMSE}}=\sqrt{\frac{1}{\mathrm{NDP}}\sum_{\mathrm{j}=1}^{\mathrm{NDP}}{({\mathrm{X}}_{\mathrm{j}}^{\mathrm{calc}.}-{\mathrm{X}}_{\mathrm{j}}^{\mathrm{exp}.})}^{2}}$$

12 $${\mathrm{AARD}}=\frac{100}{\mathrm{NDP}}\sum_{\mathrm{j}=1}^{\mathrm{NDP}}\left|\frac{{\mathrm{X}}_{\mathrm{j}}^{\mathrm{calc}.}-{\mathrm{X}}_{j}^{\mathrm{exp}.}}{{\mathrm{X}}_{\mathrm{j}}^{\mathrm{exp}.}}\right|$$

13 $${\mathrm{MAE}}=\frac{1}{\mathrm{NDP}}\sum_{\mathrm{j}=1}^{\mathrm{NDP}}\left|{\mathrm{X}}_{\mathrm{j}}^{\mathrm{calc}.}-{\mathrm{X}}_{\mathrm{j}}^{\mathrm{exp}.}\right|$$

14 $${\mathrm{RAE}}=100\times \frac{\sum_{\mathrm{j}=1}^{\mathrm{NDP}}\left|{\mathrm{X}}_{\mathrm{j}}^{\mathrm{calc}.}-{\mathrm{X}}_{\mathrm{j}}^{\mathrm{exp}.}\right|}{\sum_{\mathrm{j}=1}^{\mathrm{NDP}}\left|{\mathrm{X}}_{\mathrm{j}}^{\mathrm{exp}.}-{\bar{\mathrm{X}} }^{\mathrm{exp}.}\right|}$$

## Results and discussion

This section includes the results of relevancy analysis, ranking analysis, and a detailed investigation of determining the best model for predicting SAs solubility in ILs.

**Figure 1:**
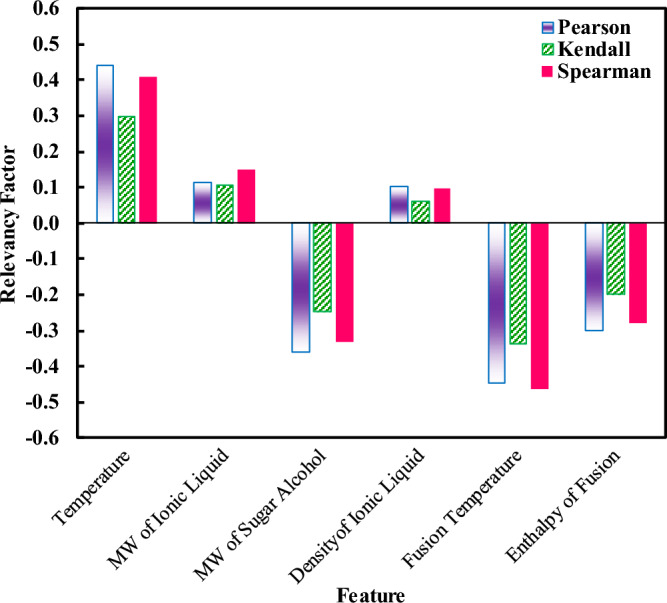
The results of relevancy analysis for the solubility of sugar alcohols in ionic liquids.

The correlation coefficients (relevancy factors) between dependent and independent variables are calculated by the 3 methods and presented in Fig. 1. To this end, 6 effective parameters, namely temperature, MWs and densities of solvents (ILs), MW of solute (SA), and the fusion temperature and enthalpy of the SA were assessed, among which temperature and fusion temperature have apparently the major impact on the solubility. The MW of SAs, as well as the enthalpy of fusion, also have a large impact on the SA solubility in ILs. The observed relevancy factors depict that while the temperature and properties of ILs (MW and density) enhance the solubility of SAs, the features of SAs (MW, fusion temperature, and enthalpy) have the opposite effect.

**Figure 2:**
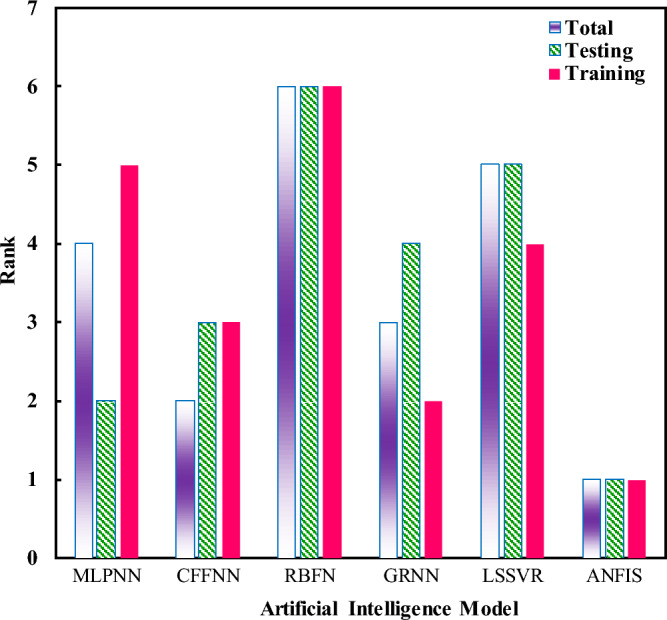
Comparing the employed neural networks based on their best performance for the solubility of sugar alcohols in ionic liquids.

The next analysis presents a ranking test, which draws a comparison among the investigated models. This is addressed in Fig. 2. It is worth noting that this comparison is made at their best structures. The features of the models’ pre-assessment, as well as the best performance of each model, are addressed in Tables 5 and 6.

**Table 5:** The features of the assessed artificial intelligence models.

Model	Model features			Number of runs
	Type	Variable	Range/type	
MLPNN	Single-layer	Number of hidden nodes	1–20	300 (15 runs per neuron)
		Activation function	Tangent hyperbolic and logarithm sigmoid	
CFFNN	Single-layer	Number of hidden nodes	1–19	285 (15 runs per neuron)
		Activation function	Tangent hyperbolic and logarithm sigmoid	
RBFNN	Single-layer	Number of hidden nodes	1–20	400 (20 spreads per neuron)
		Spread value	10^−4^–10^1^	
		Activation function	Gaussian and linear	
GRNN	Single-layer	Spread value	10^−4^–10^1^	100 (1 run per spread)
ANFIS	–	Cluster center radius (radii)	0.5–1.0	150 (5 runs per radii)
LSSVR	Linear, polynomial, and Gaussian	Kernel function	–	45 (15 runs per kernel)

**Table 6:** The best features of the assessed artificial intelligence models.

Model	Best features	Note
MLPNN	19 hidden neurons, tangent hyperbolic, and logarithm sigmoid activation functions in the hidden and output layers, respectively	Train function: Levenberg–Marquardt
CFFNN	19 hidden neurons, tangent hyperbolic, and logarithm sigmoid activation functions in the hidden and output layers, respectively	Train function: Levenberg–Marquardt
RBFNN	19 hidden neurons Gaussian and linear activation functions in the hidden and output layers, respectively Spread value = 0.5263	–
GRNN	550 hidden neurons Gaussian and linear activation functions in the hidden and output layers, respectively Spread = 0.0394	–
ANFIS	Radii = 0.435	Hybrid algorithm (combination of backpropagation scenario and least square method)
LSSVR	Gaussian kernel	Combination of simulated annealing and simplex optimization methods

Based on their performance in the training, testing, and combined phases, the models are sorted and compared in this figure. The training phase included 85% of the entire databank. To do so, the rank was calculated based on Eq. (15) and the rank indices already calculated by Eqs. (9)–(14).

15 $$\mathrm{Rank}=\mathrm{Round}\left[\frac{1}{6}\sum_{\mathrm{i}=1}^{6}{\mathrm{Rank}}_{\mathrm{Index}}(\mathrm{i})\right],\mathrm{ i}:\mathrm{R},\mathrm{ MSE},\mathrm{ RMSE},\mathrm{AARD},\mathrm{MAE},\mathrm{ and RAE}$$

Results of the ranking test clarify that the ANFIS model offers the best estimations for the solubility data for the training, testing, and entire databank, while RBFNN presents the least accuracies in the same data distribution. As a consequence, the ANFIS model is introduced as the best model and applied to simulate different SA-IL systems in the following sections. This model predicts overall experimental data with the AARD = 7.43%, MAE = 0.017, RAE = 9.28%, MSE = 0.0009, RMSE = 0.03, and R^2^ = 0.98260.

**Figure 3:**
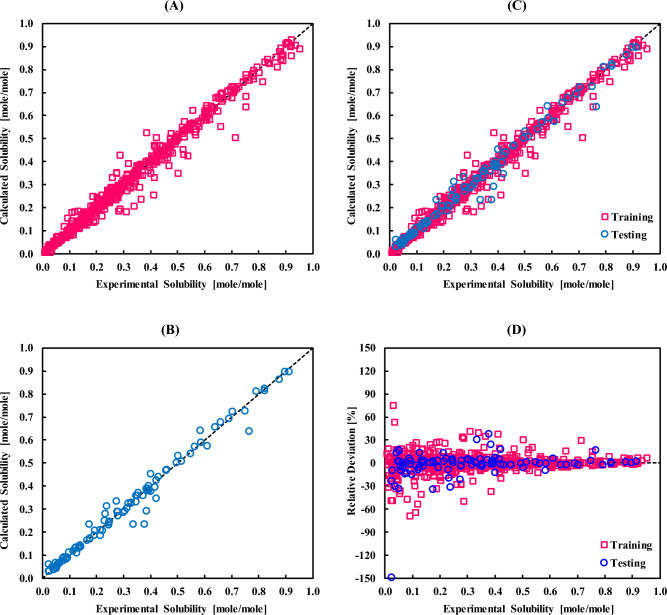
The overall performance of the ANFIS model for the dissolution of sugar alcohols in ionic liquids in terms of the calculated results in the (**A**) training, (**B**) testing, and (**C**) entire datasets and (**D**) relative deviations utilizing the ANFIS model.

Figure 3 depicts the calculated solubilities by the ANFIS model (Fig. 3A–C), as well as the relative deviations (Fig. 3D), against the experimental values. These figures approve that the calculated solubilities in the two training and testing steps are close to the real ones, which indicates the effectiveness of the model. The distribution of the relative deviations also confirms such a statement.

The observations of this figure also depict that overfitting has not occurred in this system. Indeed, when a calculation procedure tends to learn every detail of a system in the training step, and the model then acts inaccurately to estimate the testing data, overfitting has occurred. An indication of overfitting in a system is a small error on the training dataset, while large errors on the test dataset. As a consequence of overfitting, the model is not capable of generalizing the features or patterns that have already been learned in the training phase. A reason for overfitting is often the insufficient distribution of the training and testing datasets viz a small training dataset, which was not the case in this study. The employed distribution of data in this study (85% for the training set) and also the accuracies in the training and testing datasets signify that the ANFIS model did not fall into the overfitting well. This issue can be understood by tracking the residuals and standard deviations in the training and testing categories.

**Figure 4:**
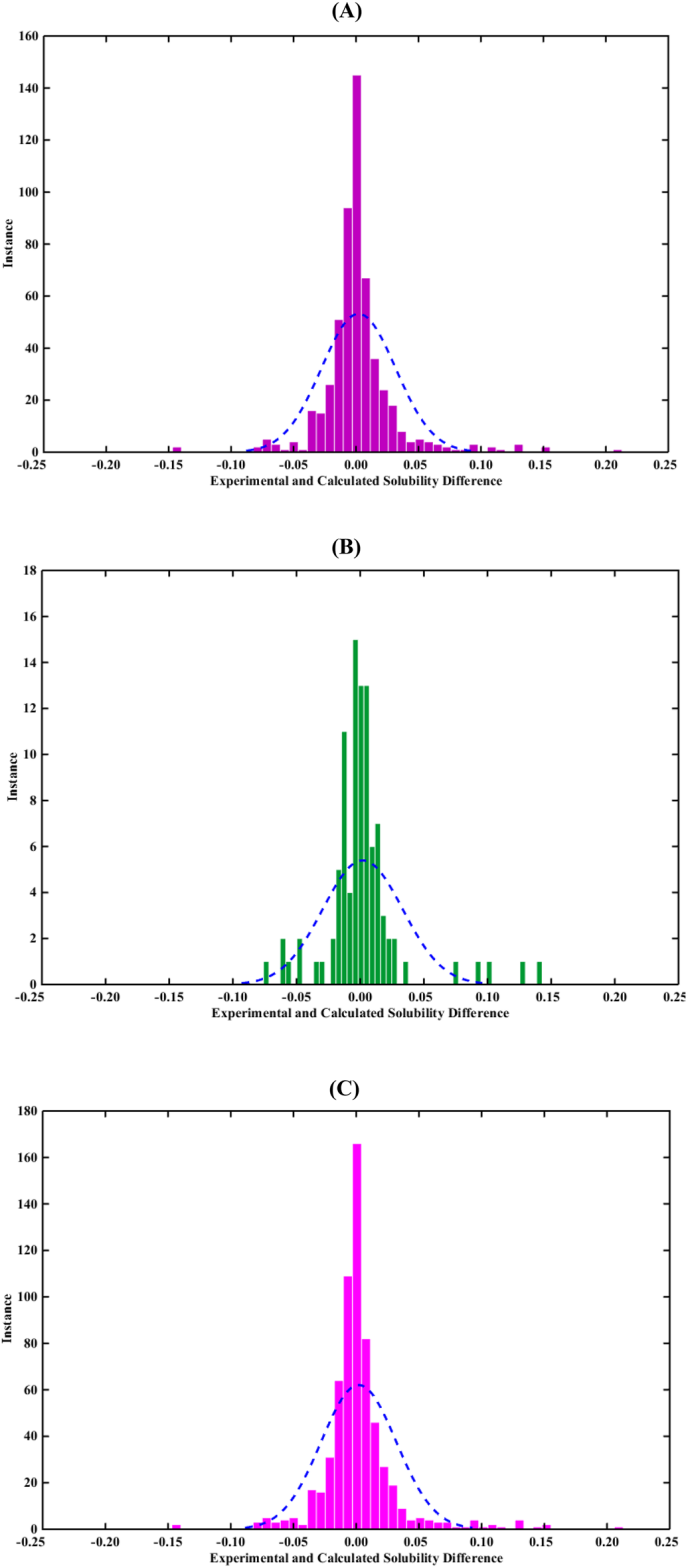
The histograms of the ANFIS deviations in the (**A**) training, (**B**) testing, and (**C**) entire datasets.

The ingredients of Fig. 4, which depict the residuals (${\mathrm{X}}_{\mathrm{i}}^{\mathrm{exp}.}-{\mathrm{X}}_{\mathrm{i}}^{\mathrm{calc}.}$) distributions indicate that those of the major portion of the dataset in training, testing, and entire datasets fall within the range of ± 0.05 (molar fraction). To this end, the average residual values and standard deviations were calculated based on Eqs. (16) and (17)^64^, respectively. The ANFIS model presents 0.0020004, 0.0019553, and 0.0019937 residuals for the testing, training, and entire datasets, while the standard deviations are 0.029708, 0.031421, and 0.029946, respectively.

16 $$\mathrm{Average \, Residual}=\frac{1}{\mathrm{NDP}}\sum_{\mathrm{i}=1}^{\mathrm{NDP}}\left({\mathrm{X}}_{\mathrm{i}}^{\mathrm{exp}.}-{\mathrm{X}}_{\mathrm{i}}^{\mathrm{calc}.}\right)$$

17 $$\mathrm{Standard \, Deviation}=\sqrt{\frac{1}{\mathrm{NDP}}\sum_{\mathrm{j}=1}^{\mathrm{NDP}}{\left[\left({\mathrm{X}}_{\mathrm{i}}^{\mathrm{exp}.}-{\mathrm{X}}_{\mathrm{i}}^{\mathrm{calc}.}\right)-\mathrm{Average \, Residual}\right]}^{2}}$$

**Figure 5:**
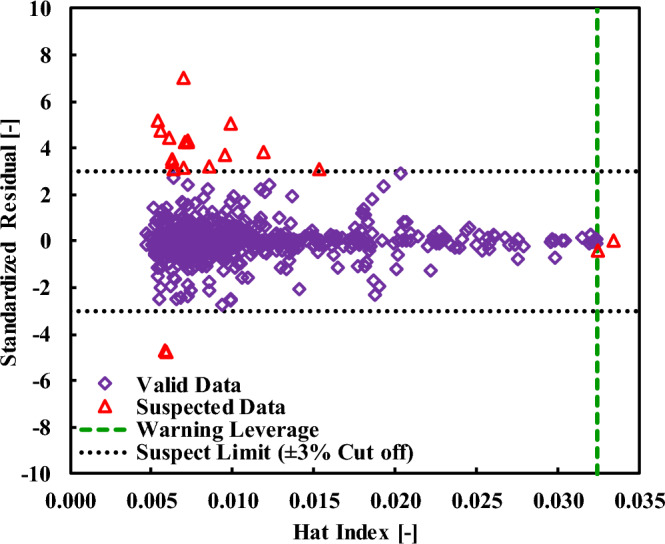
The analysis of the leverage method for detecting the valid and suspect data points for the dissolution of the sugar alcohols in ionic liquids.

A quantitative measure of the applicability of the ANFIS model, which is presented in terms of standardized residuals [Eq. (18)] and Hat Index [Eq. (19)], is addressed in Fig. 5. In Eq. (19), M is an NDP × 6 matrix showing the experimental quantities of the independent variable. Then, the leverage method can explore the region a model is applicable with the use of standardized residual information when they are in the range of ± 3. In Fig. 5, this range is identified by dotted lines. Equation (20) is utilized to determine the quantity of critical leverage.

18 $$\mathrm{Standardized \,residual \, of \, data \, point \, i}=\frac{{\mathrm{X}}_{\mathrm{i}}^{\mathrm{exp}.}-{\mathrm{X}}_{\mathrm{i}}^{\mathrm{calc}.}}{\mathrm{Standard \, deviation}};i=\mathrm{1,2},,\dots ,NDP$$

19 $$\mathrm{Hat \, Index}=\mathrm{M}{\left({\mathrm{M}}^{\mathrm{Transposed}}\times \mathrm{M}\right)}^{-1}{\mathrm{M}}^{\mathrm{Transposed}}$$

20 $$\mathrm{Critical \, leverage}=\frac{3}{\mathrm{NDP}}\left(1+\mathrm{the \,number \,of \,independent \,variables}\right)=0.0325$$

The applicability domain of the ANFIS model and the corresponding boundaries are defined in Fig. 5. In a nutshell, the leverage method confirms that the ANFIS model is readily capable of estimating the solubility of SAs in ILs based on the collected databank with high reliability. To elaborate on this, since only 20 data points among 647 solubility samples were identified as either good leverage (Hat Index > critical leverage) or outlier (standardized residuals out of the range of ± 3), the domain of applicability includes larger than 96.9% of the entire databank. On account of these findings, the ANFIS model is reliable owing to its high level of coverage and wide range of applicability.

**Figure 6:**
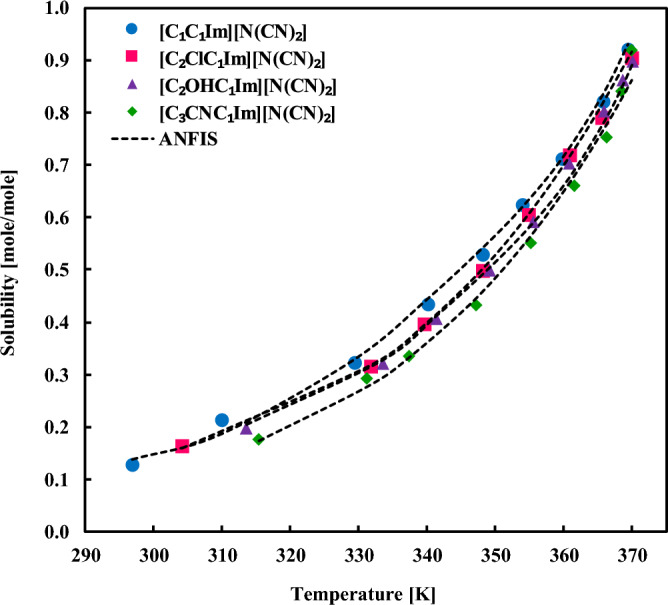
Comparing the experimental solubilities of sorbitol in varied ionic liquids with the ANFIS model’s result.

**Figure 7:**
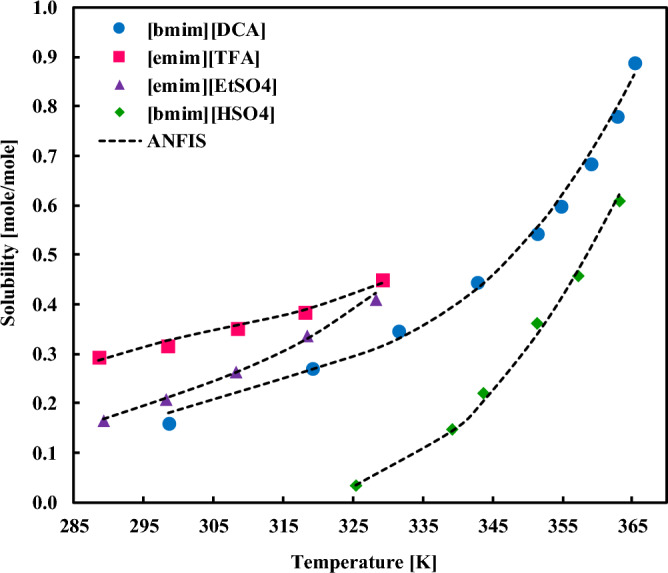
Comparing the experimental solubilities of xylitol in varied ionic liquids with the ANFIS model’s result.

**Figure 8:**
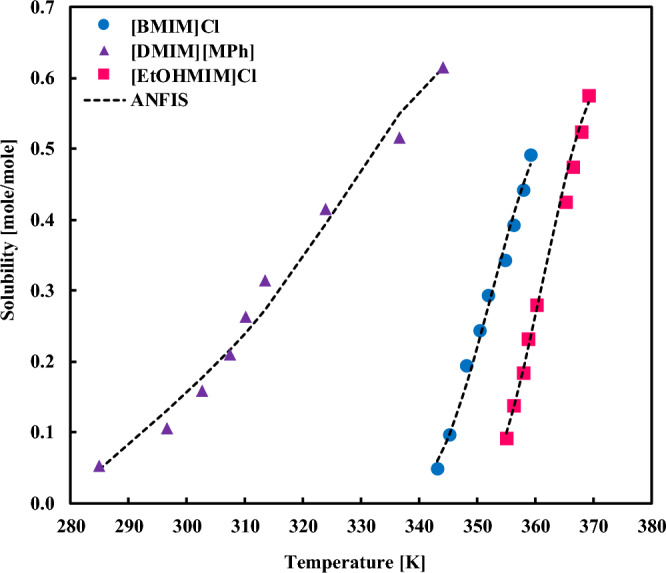
Comparing the experimental solubilities of fructose in varied ionic liquids with the ANFIS model’s result.

The solubility of sorbitol in diverse ILs is presented in Fig. 6 and compared with the ANFIS calculations. Clearly, the model can represent the solubility data in the entire range of temperatures and can also distinguish the effect of solvent type as well. Figure 7, which addresses the impact of IL on the xylitol solubility, also depicts that the ANFIS results are accurate enough in the low-to-high solubility ranges. Similar observations for the solubility of fructose in different ILs have existed in Fig. 8. It is inferred from this figure that even sharp solubility changes with the temperature can be simulated by the ANFIS model with remarkable precision.

**Figure 9:**
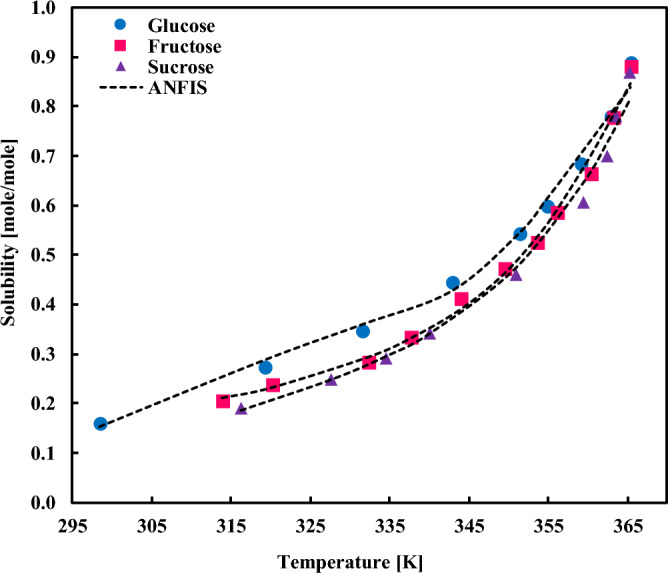
Comparing the experimental solubilities of fructose, glucose, and sucrose in [C_4_C_1_Im][CF_3_CO_2_] ionic liquid and with the ANFIS model’s result.

The solubility behavior of fructose, glucose, and sucrose in [C_4_C_1_Im][CF_3_CO_2_] IL is presented in Fig. 9, which signifies that they are well estimated by the ANFIS model within the entire range of temperatures.

**Figure 10:**
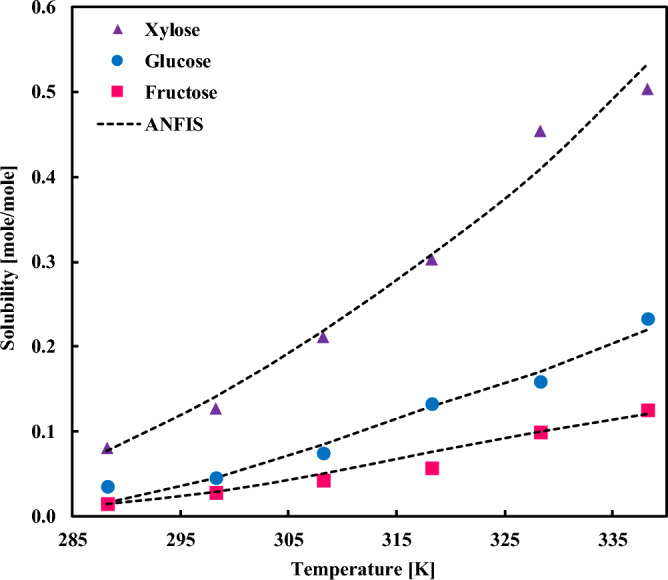
Comparing the experimental solubilities of fructose, glucose, and sucrose in [C_4_C_1_Im][(OCH_3_)_2_PO_4_] ionic liquid with the ANFIS model’s result.

[C_4_C_1_Im][(OCH_3_)_2_PO_4_] IL that offers low-to-high solubility capacity for different SA compounds, including xylose, glucose, and fructose is assessed and compared to the ANFIS estimations in Fig. 10. It can be seen that the model can describe the solubility behavior of different IL-SA pairs, from low to high solubility range, with remarkable accuracy.

**Figure 11:**
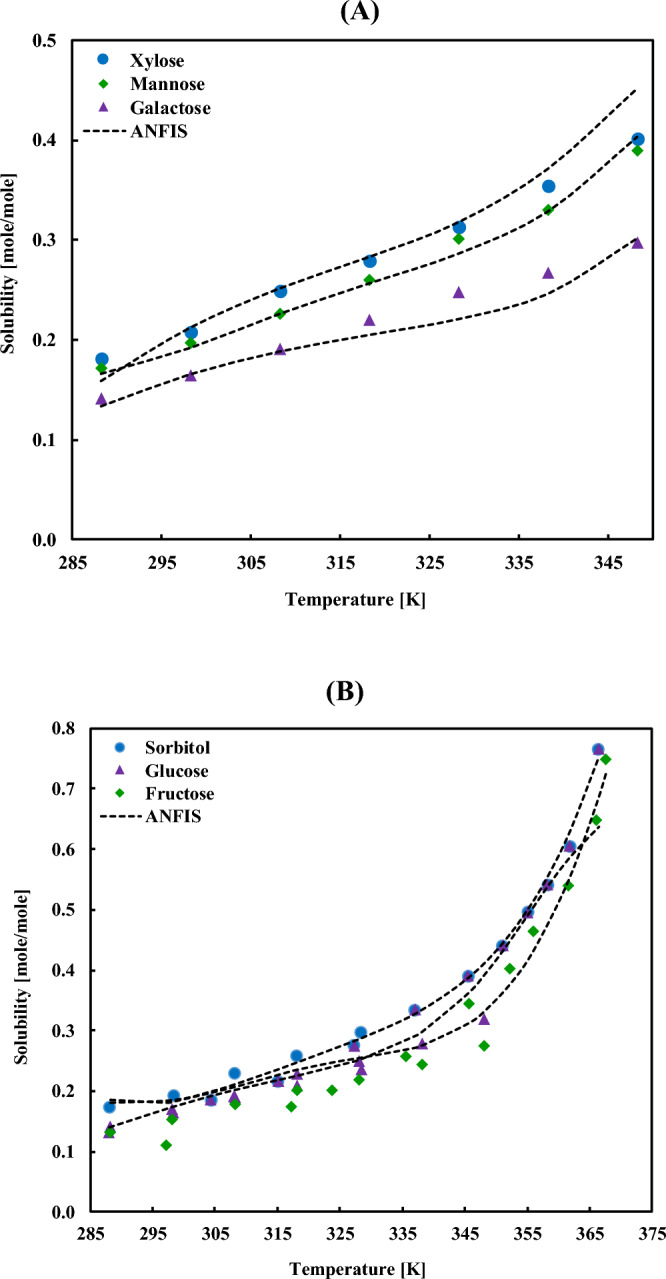
Comparing the experimental solubilities of (**A**) xylose, mannose, and galactose, and (**B**) sorbitol, glucose, and fructose in [bmim][DCA] ionic liquid with the ANFIS model’s result.

The impact of the SA compound and temperature on the absorption capacity of [bmim][DCA] IL is compared in Fig. 11. Despite some minor discrepancies in the higher solubility range, the ANFIS model can represent the real data accurately. It is worth discussing that the collected dataset and the discrepancies of different datasets also affect the accuracy of the ANFIS model and a major portion of the observed error arises from these scattering. This issue is well magnified in Fig. 11B. As per the figure, there is more than one reference for the collected data of the solubilities of sorbitol, glucose, and fructose in [bmim][DCA], and the reported quantity and even trends in each case vary to a great extent, which generates uncertainties in the model behavior.

Table 7 summarizes the performance of the ANFIS model for predicting the phase equilibrium of various SA-IL pairs. The largest deviation is observed in the case of D-xylitol solubility in [bmim][C(CN)_3_] (ARD = 9.72% and AARD = 29.62%) and the largest relative deviations (Min RD = − 28.02% and Max RD = 75.23%) belong to a data point from the same system as well. Nevertheless, the maximum AARD% does not exceed 10% in the majority of SA-IL pairs, which signifies the accuracy of the ANFIS model in representing the solubility data of SAs in ILs. The ANFIS model can be further employed for the solubility data modeling in various solutions composed of SA compounds and ILs.

**Table 7:** The accuracy of the ANFIS model’s result for different SA-IL systems in terms of various deviation factors.

Sugar alcohol	Ionic liquid	NDP	ARD%	AARD%	Min RD%	Max RD%
Xylitol	[emim][DCA]	7	− 1.67	8.82	− 11.48	17.36
Xylitol	[bmim][DCA]	14	− 0.43	4.45	− 12.64	12.25
Xylitol	[emim][TFA]	5	− 0.11	1.87	− 2.54	2.82
Xylitol	[emim][EtSO_4_]	5	− 0.17	1.43	− 2.77	2.44
Xylitol	[C_1_C_1_Im][N(CN)_2_]	8	− 0.18	0.77	− 1.33	2.00
Xylitol	[C_2_OHC_1_Im][N(CN)_2_]	11	2.24	3.85	− 3.00	15.29
Xylitol	[C_3_CNC_1_Im][N(CN)_2_]	7	− 0.24	1.33	− 2.93	2.12
Xylitol	[emim][MeOEtOEtSO_4_]	6	6.17	9.85	− 10.09	27.86
Xylitol	[bmim][HSO_4_]	6	0.87	4.22	− 5.16	7.33
Xylitol	[bmim][SCN]	6	− 0.65	6.99	− 12.26	8.27
Xylitol	[bmim][C(CN)_3_]	6	9.72	29.62	− 28.02	75.23
Sorbitol	[emim][DCA]	5	− 1.08	7.46	− 10.41	8.39
Sorbitol	[bmim][DCA]	15	− 0.31	3.72	− 8.84	8.58
Sorbitol	[emim][TFA]	5	0.32	3.08	− 5.33	3.02
Sorbitol	[emim][EtSO_4_]	4	0.60	2.11	− 1.89	5.19
Sorbitol	[C_1_C_1_Im][N(CN)_2_]	9	− 0.02	3.11	− 6.18	12.90
Sorbitol	[C_2_OHC_1_Im][N(CN)_2_]	9	− 0.13	1.77	− 3.75	3.63
Sorbitol	[C_3_CNC_1_Im][N(CN)_2_]	9	0.27	2.89	− 3.64	6.38
Sorbitol	[C_2_C_l_C_1_Im][N(CN)_2_]	8	− 0.21	1.08	− 2.60	2.41
Sorbitol	[mmim](MeO)_2_PO_2_	4	− 1.00	5.38	− 10.01	5.30
Sorbitol	[C_2_MIM][Ac]	4	2.33	4.22	− 3.18	8.63
Erythritol	[C_1_C_1_Im][N(CN)_2_]	9	− 0.11	2.38	− 4.06	4.92
Erythritol	[C_2_OHC_1_Im][N(CN)_2_]	14	0.08	1.99	− 2.75	6.63
Erythritol	[C_3_CNC_1_Im][N(CN)_2_]	10	0.16	1.04	− 2.06	3.21
Erythritol	[C_2_C_l_C_1_Im][N(CN)_2_]	10	0.31	1.59	− 4.90	4.53
Mannitol	[emim][MeOEtOEtSO_4_]	4	− 2.36	16.84	− 30.62	20.38
Mannitol	[bmim][HSO_4_]	5	− 9.13	9.15	− 32.99	0.03
Mannitol	[bmim][SCN]	6	0.75	5.18	− 13.30	8.24
Mannitol	[bmim][C(CN)_3_]	4	4.47	4.55	− 0.15	17.84
Glucose	[emim][EtSO_4_]	5	0.02	2.33	− 5.38	2.57
Glucose	[emim][TFA]	5	0.09	2.81	− 5.12	3.44
Glucose	[emim][DCA]	5	− 0.48	1.28	− 1.35	2.00
Glucose	[bmim][DCA]	22	− 0.88	5.94	− 23.22	16.81
Glucose	[EMIM][SCN]	13	− 5.62	27.42	− 69.11	32.62
Glucose	[C_4_C_1_Im][CF_3_CO_2_]	9	0.13	3.28	− 5.09	5.76
Glucose	[C_4_C_1_Im][(OCH_3_)_2_PO_4_]	6	6.70	14.01	− 14.53	53.23
Glucose	[MIMC_3_N_111_][N(CN)_2_]_2_	7	− 0.82	1.86	− 5.41	1.35
Glucose	[emim][MeOEtOEtSO_4_]	4	3.38	11.69	− 16.62	18.54
Glucose	[bmim][HSO_4_]	9	− 1.67	6.69	− 16.73	7.64
Glucose	[bmim][SCN]	5	− 4.23	23.11	− 49.14	27.20
Glucose	[bmim][C(CN)_3_]	4	− 0.57	4.66	− 4.89	8.17
Glucose	[DMIM][MPh]	9	− 0.11	3.23	− 4.68	10.11
Glucose	[BMIM]Cl	8	0.47	2.98	− 5.15	4.99
Glucose	[EtOHMIM]Cl	9	− 2.10	10.36	− 29.41	12.61
Glucose	[C_1_C_1_Im][N(CN)_2_]	5	− 0.99	4.36	− 6.13	3.74
Glucose	[C_2_OHC_1_Im][N(CN)_2_]	6	5.29	7.49	− 4.96	20.75
Glucose	[C_3_CNC_1_Im][N(CN)_2_]	6	− 0.77	6.30	− 14.90	6.95
Glucose	[C_2_C_l_C_1_Im][N(CN)_2_]	5	0.14	2.18	− 3.38	2.52
Fructose	[emim][EtSO_4_]	5	− 2.86	3.22	− 9.25	0.53
Fructose	[emim][TFA]	3	1.99	2.20	− 0.31	5.40
Fructose	[emim][DCA]	5	0.07	4.13	− 7.01	6.72
Fructose	[bmim][DCA]	22	− 2.66	22.23	− 64.12	41.24
Fructose	[EMIM][SCN]	14	− 2.77	19.23	− 49.48	34.79
Fructose	[C_4_C_1_Im][CF_3_CO_2_]	11	0.21	2.73	− 3.57	5.37
Fructose	[C_4_C_1_Im][(OCH_3_)_2_PO_4_]	6	− 9.14	10.80	− 35.74	3.24
Fructose	[MIMC_3_N_111_][N(CN)_2_]_2_	7	0.12	0.31	− 0.47	0.51
Fructose	[DMIM][MPh]	9	− 0.67	9.18	− 23.09	12.73
Fructose	[BMIM]Cl	9	− 0.17	6.80	− 22.21	14.84
Fructose	[EtOHMIM]Cl	9	− 1.31	4.72	− 8.94	7.49
Sucrose	[bmim][DCA]	8	2.85	10.66	− 11.18	27.20
Sucrose	[C_4_C_1_Im][CF_3_CO_2_]	9	0.03	3.02	− 5.89	6.65
Sucrose	[emim][MeOEtOEtSO_4_]	4	− 1.68	10.60	− 20.53	17.20
Sucrose	[bmim][HSO_4_]	6	− 1.54	6.28	− 15.66	7.10
Sucrose	[bmim][SCN]	6	− 2.65	3.74	− 11.93	3.27
Sucrose	[bmim][C(CN)3]	3	0.01	10.65	− 15.97	12.50
Sucrose	[EMIM][SCN]	14	− 3.15	20.55	− 47.36	23.59
Sucrose	[DMIM][MPh]	8	4.71	6.83	− 7.46	20.01
Sucrose	[BMIM]Cl	9	− 2.79	14.10	− 49.04	18.35
Sucrose	[EtOHMIM]Cl	9	− 16.01	27.96	− 149.07	18.40
Xylose	[emim][EtSO_4_]	5	0.06	3.07	− 4.62	3.96
Xylose	[EMIM][SCN]	6	0.41	6.29	− 17.63	10.67
Xylose	[bmim][DCA]	7	− 1.31	4.91	− 12.24	12.58
Xylose	[C_4_C_1_Im][(OCH_3_)_2_PO_4_]	6	− 1.10	5.98	− 10.87	10.18
Xylose	[emim][MeOEtOEtSO_4_]	4	− 0.19	6.86	− 9.18	13.09
Xylose	[bmim][HSO_4_]	7	0.58	4.57	− 5.57	8.07
Xylose	[bmim][SCN]	5	2.81	6.70	− 9.74	12.88
Xylose	[bmim][C(CN)_3_]	4	0.29	2.48	− 4.37	2.68
Galactose	[emim][EtSO_4_]	5	0.40	2.88	− 4.68	5.95
Galactose	[EMIM][SCN]	6	− 1.66	4.41	− 10.24	6.32
Galactose	[bmim][DCA]	7	4.56	5.08	− 1.36	10.87
Cellobiose	[EMIM][SCN]	6	0.20	7.00	− 11.89	7.86
Maltose	[EMIM][SCN]	6	− 1.32	2.68	− 11.47	3.52
Mannose	[bmim][DCA]	7	1.36	2.34	− 3.31	4.86
Mannose	[C_4_C_1_Im][(OCH_3_)_2_PO_4_]	6	1.39	5.58	− 8.88	15.91
Lactose	[EMIM][SCN]	7	0.41	9.99	− 22.72	18.53
Lactose	[DMIM][MPh]	8	− 0.35	5.12	− 9.61	7.01
Lactose	[BMIM]Cl	6	− 1.63	5.49	− 12.12	6.85
Lactose	[EtOHMIM]Cl	6	− 2.05	12.56	− 31.13	17.68
Total		647	− 0.04	7.43		

Average relative deviation percent, $\mathrm{ARD}({\%})=\frac{100}{\mathrm{NDP}}\times {\sum }_{\mathrm{i}=1}^{\mathrm{NDP}}\left(\frac{{\mathrm{X}}_{\mathrm{i}}^{\mathrm{Exp}.}-{X}_{\mathrm{i}}^{\mathrm{Cal}.}}{{X}_{\mathrm{i}}^{\mathrm{Exp}.}}\right)$.

Relative deviation percent, $\mathrm{RD}\left({\%}\right)=100\times \frac{{X}_{\mathrm{i}}^{\mathrm{Exp}.}-{X}_{\mathrm{i}}^{\mathrm{Cal}.}}{{X}_{\mathrm{i}}^{\mathrm{Exp}.}},\mathrm{ i}=\mathrm{1,2},\dots ,\mathrm{NDP}$.

Although ML studies, which consider the systems in question, cannot be for the time being found in the literature, a comparison between any available modeling approach with the one developed herein is of great interest and can then shed light on the quality of ML models. The previous calculation procedures for the solubility of SAs in ILs include thermodynamic modeling that benefits from the use of ACMs mainly including NRTL and UNIQUAC and EoS such as PC-SAFT.

To this end, PC-SAFT is a popular EoS that can benefit from either predictive or correlative schemes. Carneiro et al.^33^ developed calculations based on this method for the solubilities of xylitol and sorbitol in 1-ethyl-3-methylimidazolium dicyanamide, 1-butyl-3-methylimidazolium dicyanamide, Aliquat® dicyanamide, trihexyltetradecylphosphonium dicyanamide, and 1-ethyl-3-methylimidazolium trifluoroacetate at 288–339 K. In the whole systems investigated, they obtained 3.7–112.2% and 3.3–21.7% deviations when the predictive and correlative approaches were employed, respectively. The use of a fitting parameter in the calculations, which was determined based on the regression of the solubility data, notably improved the accuracy of calculations. Paduszynski et al.^37^ also benefitted from the same approach with some minor modifications for the system including 1-butyl-3-methylimidazolium dicyanamide 1-butyl-3-methylimidazolium trifluoroacetate ILs and glucose, fructose, and sucrose SAs. Then, they reported very poor agreement between the calculations and the measurements in the predictive mode. Benefitting from two adjustable parameters and the regression of solubility temperatures, they improved the accuracy of the model considerably. Their further study also reported the same trends^38^. Although this method of calculation was successful and is in many cases comparable to the ML calculations in this study, it demands a two-step regression procedure including the optimization of pure and binary data.

The solubilities of glucose, fructose, xylose, and galactose in two ILs namely the 1-etyhl-3-methylimidazolium ethylsulfate (also known as [emim][EtSO_4_]) and the Aliquat®336 at 288–328 K were modeled by Carneiro and co-workers^31^ by the use of NRTL and UNIQUAC equations. The AARD% obtained for the two equations did not exceed 4% (based on molar fractions) in almost all cases, and no significant difference between the two equations was observed. This team^32^ then utilized a similar methodology within the same ACMs and an e-NRTL equation for the systems composed of xylitol and sorbitol and several ILs. Their calculations based on NRTL, UNIQUAC, and e-NRTL ACMs resulted in 0.9–3.7%, 1.1–3.2%, and 0.7–2.6% deviations, respectively. Similar observations were reported in the further studies of the same research group^34^. Compared to the ML models, the thermodynamic models based on activity coefficient equations demand more sophisticated calculations as well as a regression-based procedure, which can lead to the accumulation of a large number of parameters for a vast number of SA-IL binary systems.

## Conclusions

Ionic liquids have recently been introduced to enhance the development of sugar-derived compounds and their efficient extraction. This study is the first attempt to develop several machine learning models for predicting the solubility of sugar alcohols in ionic liquids. Machine learning models were implemented using 647 solubility samples of 19 sugar alcohols in 21 ionic liquids collected from the literature. After detecting the effective variables, i.e., temperature, molecular weight and density of ILs, the molecular weight of SAs, the fusion temperature, and enthalpy, artificial neural networks, least-squares support vector regression, and adaptive neuro-fuzzy inference system (ANFIS) was appraised among which, ANFIS was the superior one. The accuracy of this model was approved by an R^2^ of 0.98359 and an AARD of 7.43% for estimating the entire databank. On the contrary, the radial basis neural network is identified as the worst model with AARD = 18.21% and R^2^ = 0.93202. Checking the ANFIS model predictions by the leverage method showed that this model is reliable because of its broad range of applicability and a remarkable level of coverage. The results of this investigation can contribute to the screening of ionic liquid solvents for the appropriate extraction of sugar alcohols. Moreover, ANFIS models can be efficiently employed for solubility estimation in the investigated SA-IL systems.

## Supplementary Information

Supplementary Information.

## Data Availability

All the collected data from the literature which are analyzed in the present study is added to the manuscript (please see Supplementary Information).
